# Perianal panniculitis presenting as chronic perianal pain: a diagnostic challenge

**DOI:** 10.1093/jscr/rjaf340

**Published:** 2025-05-30

**Authors:** Imran Thariq Ajmal, Amrithraj Thiyagarajan, Felix Anand Raj, Santhya Govindaraju, P Pravali, Venkiteswaran Muralidhar

**Affiliations:** Department of General Surgery, Chettinad Hospital and Research Institute, Chettinad Academy of Research and Education, OMR, Chennai, Pin 603103, India; Department of General Surgery, Chettinad Hospital and Research Institute, Chettinad Academy of Research and Education, OMR, Chennai, Pin 603103, India; Department of General Surgery, Chettinad Hospital and Research Institute, Chettinad Academy of Research and Education, OMR, Chennai, Pin 603103, India; Department of General Surgery, Chettinad Hospital and Research Institute, Chettinad Academy of Research and Education, OMR, Chennai, Pin 603103, India; Department of General Surgery, Chettinad Hospital and Research Institute, Chettinad Academy of Research and Education, OMR, Chennai, Pin 603103, India; Department of Bioinformatics, University of Birmingham, Birmingham, United Kingdom

**Keywords:** perianal pain, inflammation, histopathological examination

## Abstract

Perianal panniculitis is a rare inflammatory condition characterized by painful, tender, and swollen lesions in the perianal region. It can mimic other conditions such as abscesses, fistulas, malignancies, or inflammatory bowel disease. This case reflects on the rarity of Perianal panniculitis and its varied presentations and the importance of considering it in the differential diagnosis of perianal lesions. Furthermore, the case highlights limited literature addressing tools of diagnosis, management, and protocol for long-term follow-up to ensure early identification and appropriate management in case of recurrence. Considering perianal panniculitis in the differential diagnosis is crucial to avoid misdiagnosis and ensure timely treatment.

## Introduction

Perianal pain is a common clinical complaint with a broad differential diagnosis, ranging from benign conditions like hemorrhoids to more complex inflammatory or neoplastic processes. While abscesses, fistulas, and inflammatory bowel disease (IBD) are well-recognized causes, perianal panniculitis remains a rare and often underdiagnosed entity [1, 2].

Panniculitis refers to inflammation of subcutaneous fat, which can occur due to infection, trauma, autoimmune conditions, or metabolic disorders. When it affects the perianal region, it can mimic more common conditions, leading to delays in diagnosis and treatment. Clinical presentations are often nonspecific, including localized pain, swelling, and tenderness, which overlap with other perianal disorders [1]. Due to its rarity and diagnostic complexity, perianal panniculitis is not always considered in the initial evaluation, often requiring histopathological confirmation. Recognizing this condition is essential for guiding appropriate management and avoiding unnecessary or ineffective treatments. This case highlights the diagnostic challenges and clinical significance of perianal panniculitis, emphasizing the need for increased awareness among clinicians [2].

## Case report

A man in his 40s presented to our surgical unit with a 3-month history of perianal pain, which he described as an intermittent pricking sensation, worsening after bowel movements. The pain was aggravated by consuming spicy food and had no identifiable relieving factors. He also reported a lumpy sensation in the perianal region but denied rectal bleeding, prolapsing masses, burning urination, or constipation. There was no history of weight loss or systemic symptoms such as fever or night sweats. The patient had no known comorbidities, and his medical, surgical, and family histories were unremarkable. He was not on any long-term medications, did not smoke, and reported occasional alcohol consumption. There was no personal or family history of IBD, connective tissue disorders, or dermatological conditions. The patient had been treated elsewhere conservatively for close to a 2 months duration.

### Clinical examination

On general and systemic examination, the patient appeared well, with no signs of systemic illness. His vital signs were within normal limits. A digital rectal examination revealed an increased anal tone with a skin tag at the 6 o’clock position. An extra-luminal nodular growth was felt at the 11 o’clock position, which was firm, irregular, and nontender of size 3 × 2 cm on the right side extending from the right gluteal cleft. The mucosa was freely mobile over the growth. There were no external or internal fistulous orifices and no evidence of fissures, external hemorrhoids, or rectal bleeding. The examination glove was stained with feces. Anoscopy was done and no fissure or internal hemorrhoids, mass, polyp were seen.

## Investigations

### Laboratory investigations

The total leukocyte count was 16 000/mm^3^ and suggested an inflammatory process. Glycated hemoglobin (HbA1c): 5.1% which was within normal limits, ruled out poorly controlled diabetes as a contributing factor. Renal function tests were normal. Urinalysis was normal and stool occult blood was negative, so no gastrointestinal bleeding or malignancy was suspected.

Inflammatory markers were normal (ESR 10 mm/h, CRP less than 10 mg/dl, fecal calprotectin values were within 50 μg).

### Radiological imaging

**Figure 1:**
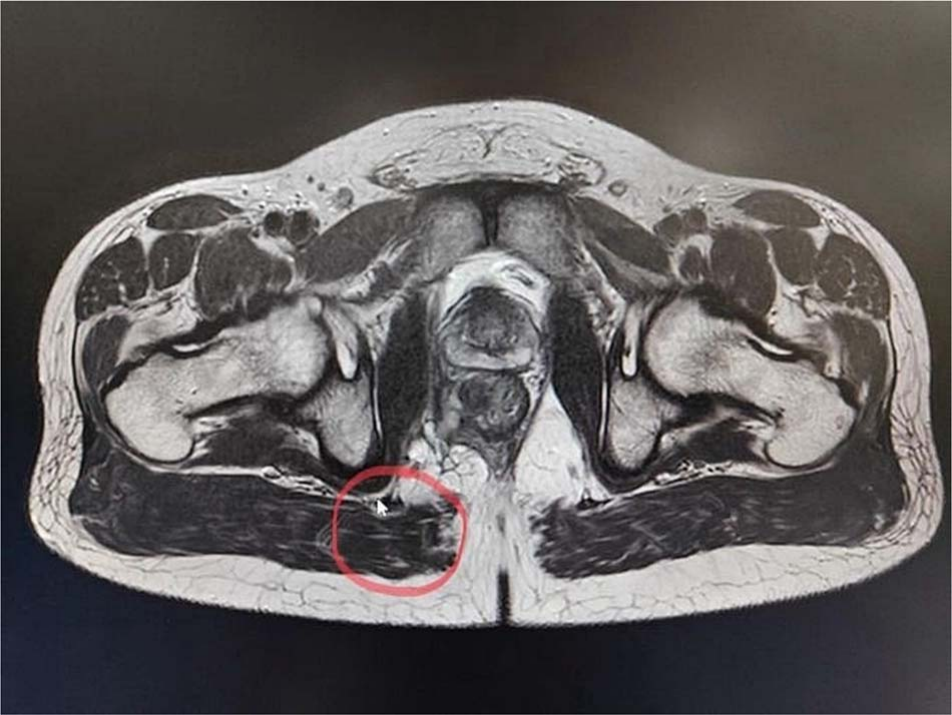
A possible fistulous tract was observed in the right perianal region, with a distal blind end in the intergluteal cleft. The fistulous tract does not involve the anal sphincter complex/levator ani muscle.

**Figure 2:**
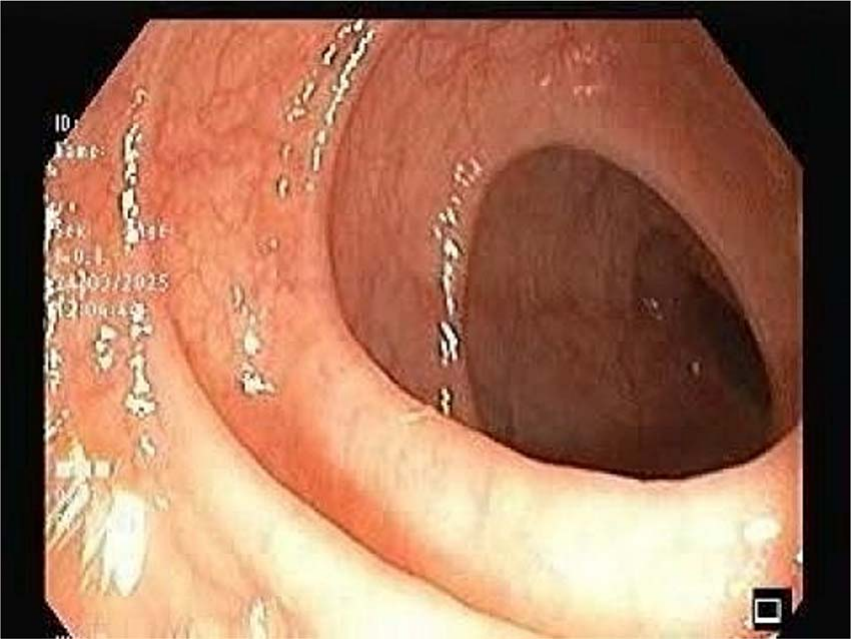
The image depicting normal colonic mucosa up to caecum with normal vascular pattern.

**Figure 3:**
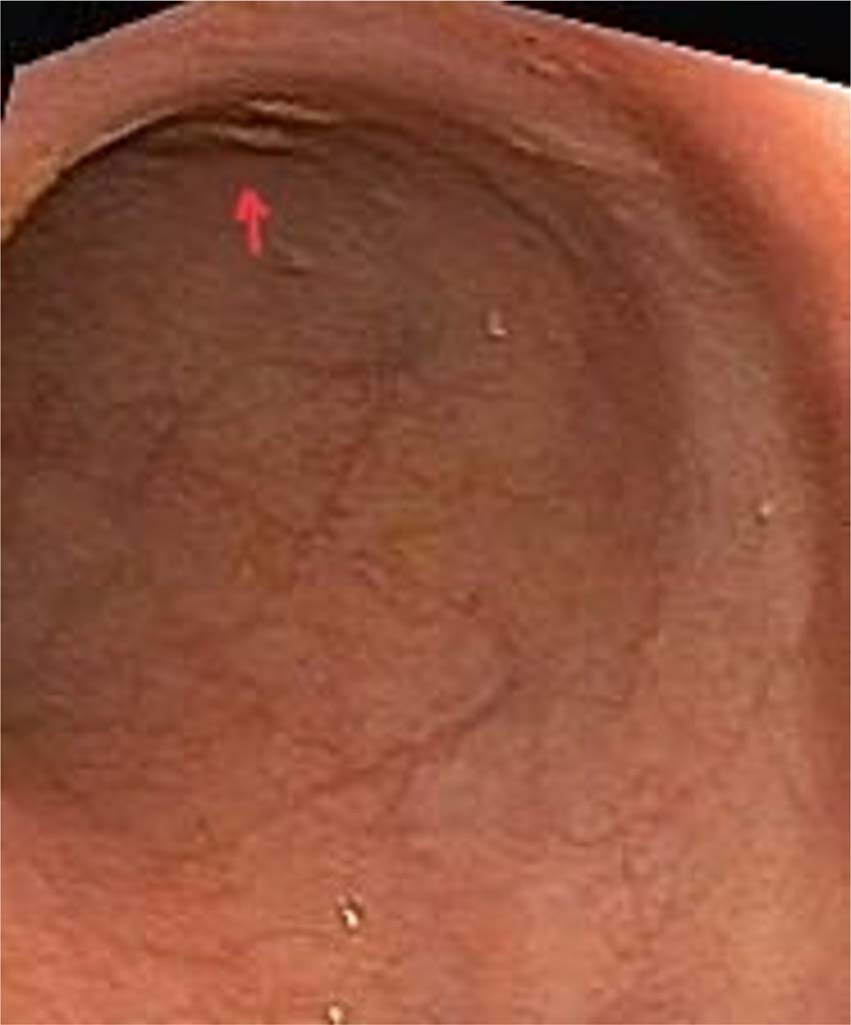
Picture depicting normal rectal mucosa with few tiny erosions.

CT scan of the pelvis suggested a chronic inflammatory process. An MRI Perianal Region/Fistulogram (Fig. 1) was done and the findings were as follows: A possible fistulous tract was observed in the right perianal region, with a distal blind end in the intergluteal cleft. The presence of a fistulous tract and its extent was unclear. MRI findings could not differentiate between infectious, inflammatory, or neoplastic processes but it can definitely point out the presence of an abscess, which was not the case in our patient. Owing to the difficulty in establishing the diagnosis a colonoscopy was done and the findings (Figs 2 and 3) were as follows: A semicircumferential area from 7 to 11 o’clock position with tiny erosions was noted, which extended 4 cm from the anal verge.

No ulcerations, strictures, or masses suggestive of malignancy were detected. Endoscopic biopsies from the area suggested no specific pathology. The colonic mucosa appeared normal beyond the affected area, making systemic inflammatory disease less likely.

## Treatment

The patient was managed with a combination of surgical intervention, pharmacological therapy, and supportive care.

### Surgical intervention

**Figure 4:**
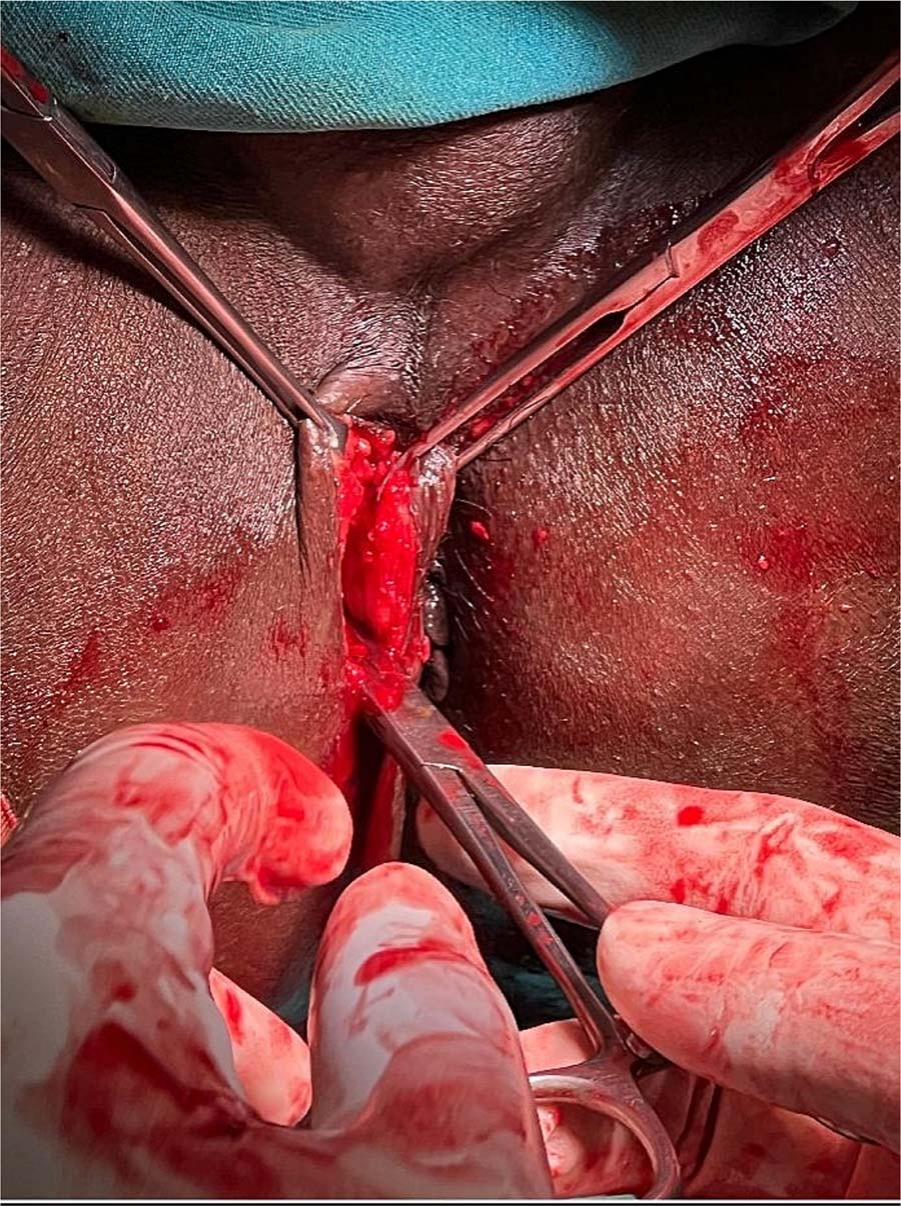
Intraoperative picture depicting excision and biopsy via perianal route.

**Figure 5:**
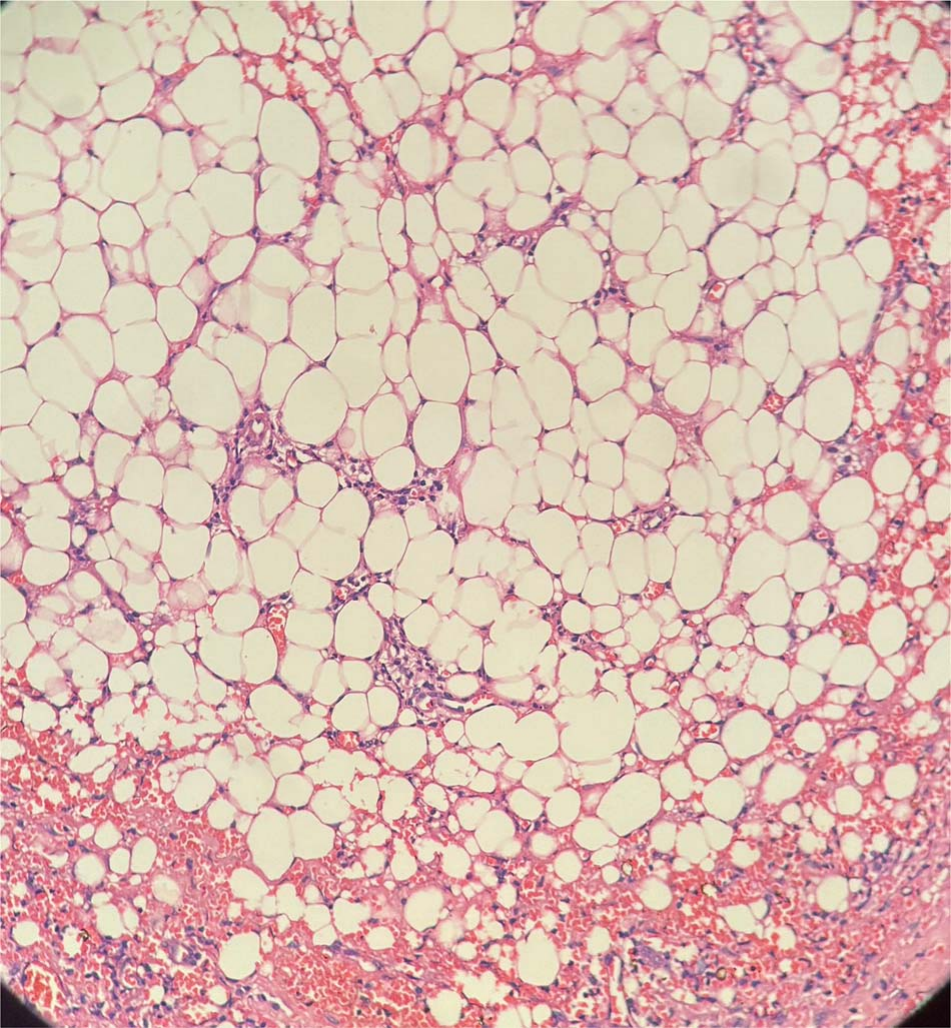
Picture depicting evidence of fat necrosis in the excised perianal tissue.

**Figure 6:**
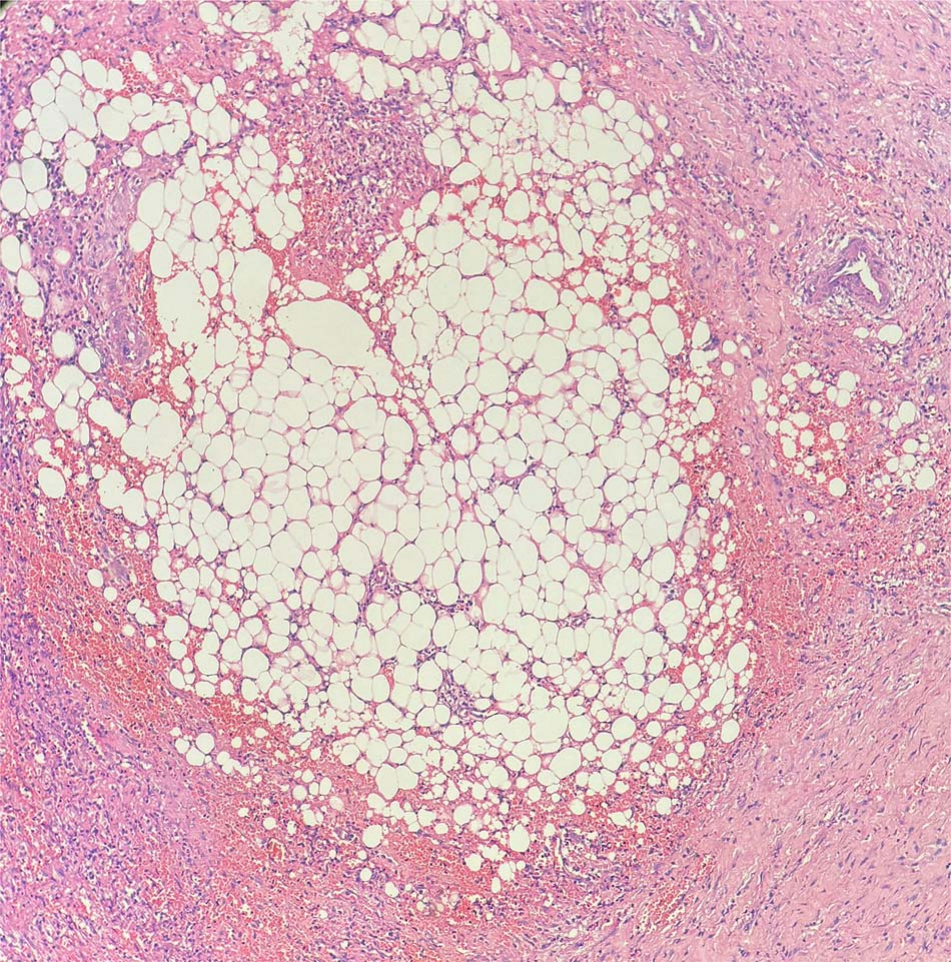
Sections show a dense inflammatory infiltrate within the subcutaneous adipose tissue, consistent with panniculitis. The infiltrate is composed of neutrophils, lymphocytes, plasma cells, and histiocytes.

**Figure 7:**
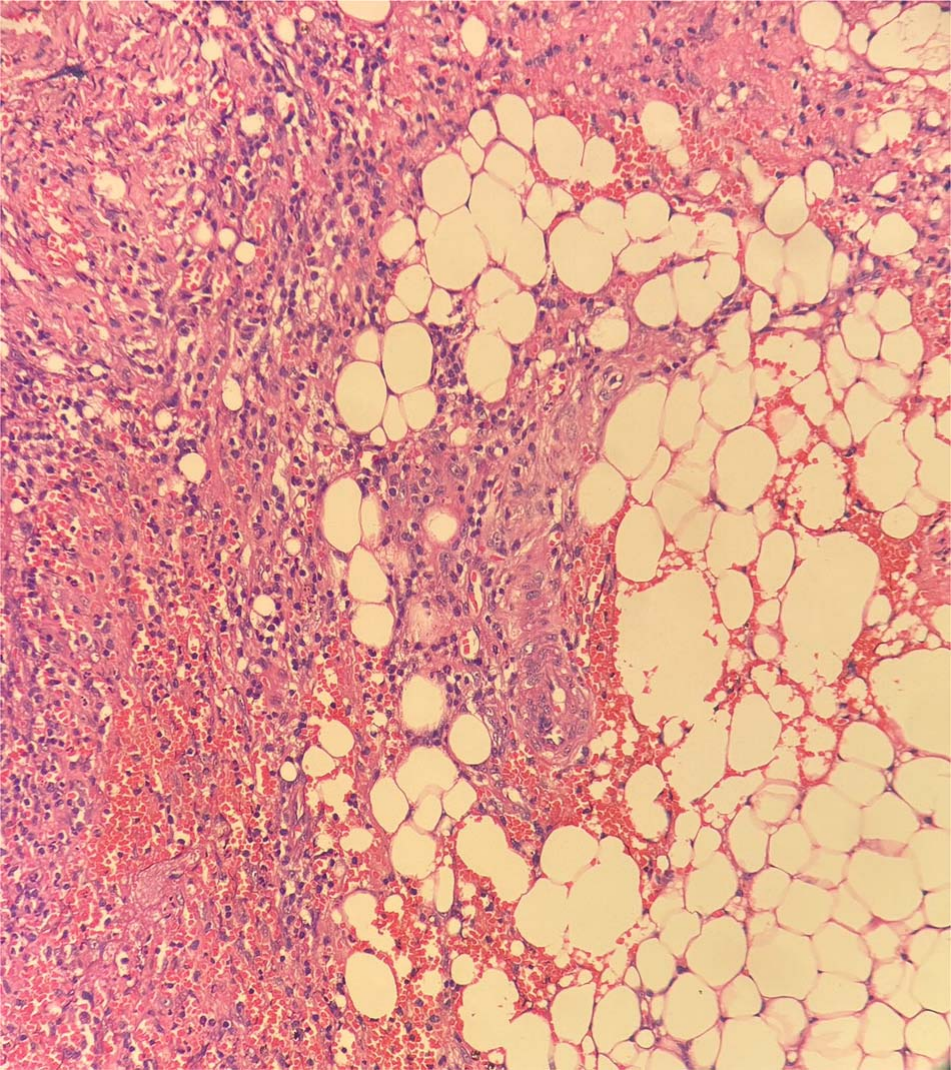
Histopathological examination showed ghost-like adipocytes and foamy histiocytes.

The patient underwent examination under anesthesia and exicision and biopsy (Fig. 4) of the extra-luminal growth via perianal route under spinal anesthesia. A curvilinear incision was made in the right perianal region from 7 o’ clock to 11 o’clock position, 1 cm away from the anal verge to access the tissue, which was then excised in toto and sent for histopathological examination. The cavity was left open to heal by secondary intention. Histopathological examination (Figs 5–7) which was a definitive diagnosis revealed features suggestive of panniculitis. This was the key diagnostic test, confirming perianal panniculitis rather than a fistula or malignancy.

Surgery was performed primarily for diagnostic and symptomatic relief purposes, as excision of inflamed tissue can reduce local inflammation and prevent further complications such as abscess formation.

### Pharmacological treatment

Postoperatively, the patient was managed with Intravenous antibiotics and analgesics. Topical antibiotic ointments are applied twice daily to the perianal wound to prevent secondary infections and aid healing. Sitz bath with warm saline water was given twice daily to reduce inflammation and promote local wound healing. A high-fiber diet and stool softeners were advocated to ensure painless defecation and prevent local trauma with regular wound care and hygiene education to prevent the recurrence of inflammation.

## Outcome and follow-up

The patient had an uneventful recovery. He was discharged on postoperative day 5 with instructions for daily sitz bath, topical antibiotic ointment application, and dietary modifications to facilitate painless defecation. At his 2-week follow-up, he reported significant pain relief, no recurrent perianal discomfort, and no signs of infection or wound complications. By 1 month postsurgery, he was asymptomatic, had resumed normal daily activities, and had a well-healed surgical site. At 3 months postsurgery, he remained symptom-free, with no recurrence of perianal pain, swelling, or discharge and the cavity completely healed. He was advised to monitor for recurrence and report any new perianal symptoms. Currently, the patient remains alive and in good health, with no recurrence of symptoms at his most recent follow-up. Routine wound care and dietary modifications continue to be part of his management, and no further interventions have been required. Long-term follow-up is essential to monitor for recurrence, particularly given the potential for chronic inflammatory conditions to resurface.

## Discussion

Perianal panniculitis is a rare inflammatory condition of the subcutaneous fat in the perianal region. While panniculitis can occur in various anatomical locations, its occurrence in the perianal region is particularly uncommon and presents unique diagnostic and management challenges. Histologically, perianal panniculitis can present as globular, septal, or mixed panniculitis, often with varying degrees of vasculitis or fat necrosis.

### Clinical presentation

Patients typically present with erythematous, tender nodules or plaques in the perianal region, sometimes associated with ulceration or drainage. The differential diagnosis includes perianal abscesses, hidradenitis suppurativa [2], cutaneous schistosomiasis [3, 4], Crohn’s disease, and infectious processes such as tuberculosis or syphilis. Panniculitis of mesentery is also a possibility, which is often misdiagnosed as acute diverticulitis [5]. Biopsy is often necessary to distinguish panniculitis from other perianal inflammatory conditions and to identify specific histopathological features, including fat necrosis, inflammatory infiltrates, and possible vasculitis.

Panniculitis tends to present as tender nodules in the perianal skin rather than deep ulcerations, fistulas, or abscesses [6].

## Conclusion

Conservative management is the first line treatment in established perianal panniculitis, but in refractory cases such as ours, surgery seems to be the best option of treatment, as far as relief of symptoms is concerned. Thus highlighting the importance of close follow-up in rare perianal conditions to ensure early identification and intervention if symptoms recur.

Hence, we would like to present panniculitis, an often underdiagnosed condition to be considered in the differentials of chronic perianal pain.
